# Ebselen, Iron Uptake Inhibitor, Alleviates Iron Overload-Induced Senescence-Like Neuronal Cells SH-SY5Y via Suppressing the mTORC1 Signaling Pathway

**DOI:** 10.1155/2023/6641347

**Published:** 2023-09-12

**Authors:** Sirirak Mukem, Ibrahim Sayoh, Saowanee Maungchanburi, Tipsuda Thongbuakaew

**Affiliations:** ^1^School of Medicine, Walailak University, Nakhon Si Thammarat 80160, Thailand; ^2^Department of Anatomy, Faculty of Science and Technology, Princess of Naradhiwas University, Narathiwat 96000, Thailand; ^3^Department of Biomedical Sciences and Biomedical Engineering, Faculty of Medicine, Prince of Songkla University, Songkhla 90110, Thailand

## Abstract

Increasing evidence highlights that excessive iron accumulation in the brain plays a vital role in neuronal senescence and is implicated in the pathogenesis of age-related neurodegenerative diseases, including Alzheimer's disease (AD) and Parkinson's disease (PD). Therefore, the chemical compounds that eliminate an iron overload may provide better protection against oxidative stress conditions that cause the accumulation of senescent cells during brain aging. Ebselen has been identified as a strongly useful compound in the research on redox biology mechanisms. We hypothesized that ebselen could alleviate an iron overload-induced oxidative stress and consequently reverses the senescence-like phenotypes in the neuronal cells. In the present study, SH-SY5Y cells were treated with ferric ammonium citrate (FAC) before ebselen, and the evaluation of the cellular iron homeostasis, the indicators of oxidative stress, and the onset of senescence phenotypes and mechanisms were carried out accordingly. Our findings showed that ebselen ameliorated the FAC-mediated iron overload by decreasing the expression of divalent metal transporter 1 (DMT1) and ferritin light chain (FT-L) proteins. In contrast, it increased the expression of ferroportin 1 (FPN1) protein and its correlation led to a decrease in the expression of the cytosolic labile iron pool (LIP). Furthermore, ebselen significantly reduced reactive oxygen species (ROS) and rescued the mitochondrial membrane potential (ΔΨm). Notably, ebselen restored the biomarkers of cellular senescence by reducing the number of senescence-associated *β*-galactosidase (SA-*β*-gal) positive cells and senescence-associated secretory phenotypes (SASP). This also suppressed the expression of p53 protein targeting DNA damage response (DDR)/p21 cyclin-dependent kinase (CDK) inhibitor through a mTORC1 signaling pathway. Potentially, ebselen could be a therapeutic agent for treating brain aging and AD by mitigating iron accumulation and restoring senescence in SH-SY5Y cells.

## 1. Introduction

Iron is an essential trace element for normal brain physiological functions such as oxygen transportation, cellular metabolism, myelination, and neurotransmitter synthesis [1]. Iron is transported from the circulatory system into the brain parenchymal tissue through the blood-brain barrier (BBB), primarily mediated by either the transferrin/transferrin receptor (Tf/TfR) or the divalent metal transporter 1 (DMT1) pathways. These mechanisms predominantly take place within the brain microvascular epithelial cells (BMVECs) [2, 3]. Neurons acquire iron through DMT1, a key player in a nontransferrin-bound iron (NTBI), which facilitates ferrous iron (Fe^2+^) uptake from the plasma membrane and its subsequent transportation from endosomes into the cytosolic labile iron pool (LIP). Notably, this process is altered within the context of the aged brain [4]. However, the accumulation of excessive iron in the brain can initiate the Fenton reaction, resulting in the generation of hydroxyl free radicals. Given the relatively lower levels of antioxidant defenses, this process can trigger downstream events that eventually lead to cell death [5].

Brain iron overload is the major function of aging and plays an important role in the pathogenesis of age-related neurodegenerative diseases, including Alzheimer's disease (AD) and Parkinson's disease (PD), although the molecular mechanisms underlying iron-induced neurodegeneration remain an ongoing examination [6]. Hence, the maintenance of cellular iron homeostasis must be tightly regulated by a multitude of iron uptake (TfR and DMT1), iron storage (ferritin), iron export (ferroportin 1; FPN1), and its ligand, the master iron-regulatory hormone hepcidin. These mechanisms concomitantly occur to ensure the normal physiological levels of cytosolic LIP for the cellular requirements and permit the safe handling of iron in the brain [6–8]. Recent studies have demonstrated that the increase in the NTBI pool is coupled with the neuroinflammation response to proinflammatory cytokines release and is considered to increase brain aging and several neurodegenerative diseases [9]. The mammalian target of rapamycin (mTOR) is an evolutionarily conserved Ser/Thr protein kinase that belongs to the phosphatidylinositol-3 kinase- (PI3K-) related kinase family. The mTOR is present as a catalytic subunit in two protein complexes: mTOR complex 1 (mTORC1) and mTOR complex 2 (mTORC2) [10, 11]. The mTOR pathway is a central regulator of cellular metabolism and physiology, with important roles in cell growth and proliferation [12, 13]. Although the involvement of the mTOR pathway in regulating cellular senescence and aging has been studied extensively, the mechanism through which this occurs is still unclear. Furthermore, many of the processes controlled by mTORC1 are dependent on iron as a cofactor and would require regulation of iron homeostasis [14]. However, the activation of the mTOR pathway is considered to be the driving force in AD pathogenesis. To date, the mechanism through which the downregulation of mTOR signaling triggers the degradation of amyloid-*β* and prevents the further accumulation of tau protein has been revealed [15]. As a result, targeting the inhibition of mTOR has been widely employed in age-related diseases to slow down the progression of AD [16].

Senescent cells undergo a phenotype development that extends beyond growth inhibition, characterized by irreversible cell cycle arrest with distinct phenotypic alterations and molecular changes within the cells [17]. Common features arising in senescent cells are the enhanced enzymatic activity of the lysosomal hydrolase senescence-associated *β*-galactosidase (SA-*β*-gal) and cell cycle checkpoint protein p53, which involve the inhibition of cyclin-dependent kinases p21 (CDKN1A) activated by DNA damage response (DDR) and is one of the major signaling pathway towards mitochondrial dysfunction [17, 18]. It is well established that p53 acts as an upstream regulator of mTOR signaling, which highlights the mechanisms underlying the regulation of iron homeostasis and cellular senescence [19]. One of the most important activities of senescent cells is developing a complex of senescence-associated secretory phenotype (SASP). Its prolonged secretion reinforces the spread of premature senescence to neighboring cells and may contribute to the earlier stage of neurodegenerative disease [20]. In addition, a strong correlation between senescence phenotypes and AD patient brains has been documented in a transcriptomic analysis study [21]. Previous *in vitro* studies have demonstrated that treating cells with ferric ammonium citrate (FAC) can induce cellular senescence due to iron dysregulation [22, 23]. Conversely, these senescent cells exhibit resistance to iron-induced ferroptosis [23]. Therefore, using synthetic iron inhibitor compounds as novel tools in the treatment of cell senescence and highlighting the efficacy of these agents, are the critical steps for neurodegenerative disease interception.

Ebselen (2-phenyl-1,2-benzisoselenazol-3(2H)-one) is a synthetic organoselenium compound first described in redox biology as a potent antioxidant [24]. It is a well-known mimicking glutathione peroxidase (GPx) that degrades hydrogen peroxide (H_2_O_2_) and organic peroxides in the brain [24]. Ebselen has been studied extensively in clinical trials to limit the neurological deficits that occur in ischemic stroke [25], bipolar disorders [26], and AD [27]. Previous studies reported that ebselen is a potent inhibitor of DMT1-mediated Fe^2+^ uptake, limiting iron-catalyzed cellular damage [28–30]. To date, ebselen has not been widely used as a therapeutic agent for reducing senescent-like neuronal cells caused by iron overload-mediated brain aging.

We speculate that DMT1 iron uptake might be an early target molecule promoting brain iron dyshomeostasis caused by ferric ammonium citrate (FAC). Therefore, the aims of this study were to determine whether ebselen could alleviate and counteract the iron accumulation-induced senescence in SH-SY5Y cells. The findings may potentially provide an insight into the therapeutic effect of ebselen to counteract iron burden and cellular senescence via its antiaging effect.

## 2. Materials and Methods

### 2.1. Reagents and Antibodies

Ebselen, ferric ammonium citrate (FAC), and anti-actin were purchased from Sigma-Aldrich (St. Louis, MO, USA). Anti-DMT1 was obtained from EMD Millipore (Rahway, NJ, USA). Anti-mTORC1 and anti-p-mTORC1 were obtained from Cell Signaling Technology, Inc. (Danvers, MA, USA). Anti-FPN1, anti-p53, and anti-p21 were purchased from Abcam (Cambridge, CB2 0AX, UK). Anti-FT-L and cell culture reagents were obtained from Thermo Fisher Scientific Inc. (Waltham, MA, USA). All chemicals used in this study were of analytical grade.

### 2.2. Cell Culture and Treatments

Human neuroblastoma cell line SH-SY5Y was obtained from the American Type Culture Collection (ATCC, Manassas, VA, USA; CRL-2266). Cells were cultured in Dulbecco's Modified Eagle Medium/Nutrient Mixture F-12 (DMEM/F12) containing 10% heat-inactivated fetal bovine serum (FBS) and 1% penicillin/streptomycin at 37°C in a 5% CO_2_ humidified incubator. Cells were then cultured until the monolayers reached 80% confluence. The passages from the 3^rd^ to the 6^th^ of cells were used for subsequent experiments.

**Figure 1:**
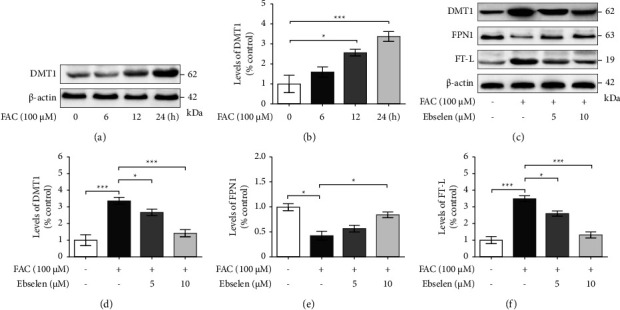
Ebselen attenuated changes in iron regulation following FAC-induced iron overload in SH-SY5Y cells. Cells were exposed to 100 *μ*M FAC for 0, 6, 12, and 24 h. (a) Western blot analysis of the DMT1 in cells exposed to 100 *μ*M FAC at different times and (b) the quantitative analysis of the protein expression normalized using *β*-actin. Cells were exposed to FAC (100 *μ*M) for 24 h prior to ebselen (5 and 10 *μ*M) for 24 h. (c) Western blot analysis of the DMT1, FPN1, and FT-L, and the quantitative analysis of the (d) DMT1, (e) FPN1, and (f) FT-L protein expression were normalized using *β*-actin, respectively. The data are the mean ± SEM of the three independent experiments performed in triplicate. ^*∗*^*P* < 0.05 and ^*∗∗∗*^*P* < 0.001 vs. the untreated group. ^*∗*^*P* < 0.05 and ^*∗∗∗*^*P* < 0.001*vs*. the FAC alone group (see supplementary data 1-2 for the original image of Figure 1(a) and supplementary data 3–6 for the original image of Figure 1(c), respectively).

Once the cells reached approximately 80% confluence, cells were seeded into culture plates. After an overnight serum deprivation, 100 *μ*M FAC in the serum-free medium was applied to the cells for 24 h. Thereafter, the different concentrations of ebselen in serum-free medium (5 and 10 *μ*M) were applied for an additional 24 h. The culture duration for FAC treatment was based on the preliminary time-course experiments, that increased the susceptibility of the expression of iron importer DMT1 protein (see Figures 1(a) and 1(b)).

### 2.3. Cell Viability Assay

The cell viability was measured using a 3-(4,5-dimethylthiazol-2-yl)-2,5-diphenyltetrazolium bromide (MTT) colorimetric assay (Sigma-Aldrich, St. Louis, MO, USA). Briefly, SH-SY5Y cells were seeded into 96-well plates at a density of 2 × 10^4^ cells/well. Cells were then treated with 100 *μ*M FAC for 24 h before the addition of the various concentrations of ebselen (0, 3, 5, 10, and 20 *μ*M) and incubated for 24 h. These were followed by the inclusion of 2 mg/ml MTT solution in each well and the 2-hour incubation in an incubator. The MTT solution was then completely removed and dimethyl sulfoxide (DMSO) was added to dissolve the purple formazan crystals. The amount of MTT formazan product was measured at 570 nm using the Synergy HT microplate reader (BioTek, Winooski, VT, USA). Cell viability was expressed as a percentage relative to the untreated control.

### 2.4. Intracellular Calcein-Chelatable Iron Assay

The levels of the cytosolic labile iron pool (LIP) were estimated using the metal-sensitive fluorophore calcein-AM [31] from Invitrogen (Carlsbad, CA, USA) according to the manufacturer's instructions. Briefly, SH-SY5Y cells were seeded into 96-well plates at a density of 2 × 10^4^ cells/well. Cells were then treated with 100 *μ*M FAC for 24 h before the addition of ebselen (5 and 10 *μ*M) and incubated for 24 h. Cells were subsequently washed twice with 1X phosphate-buffered saline (PBS) and incubated with 1 *μ*M calcein-AM at 37°C for 30 min. The excessive calcein-AM on the cell surface was gently washed twice with 1X PBS followed by the measurement of the fluorescence intensity using the Synergy HT microplate reader (BioTek, Winooski, VT, USA) at excitation/emission wavelengths of 485/538 nm. The images of the cells were acquired immediately and analyzed by using a fluorescence microscope (ZEISS, Germany).

### 2.5. Measurement of Intracellular Reactive Oxygen Species (ROS)

The intracellular ROS levels were assessed using the cell-permeable reagent fluorogenic probe, 2′-7′dichlorofluorescin diacetate (DCFDA) from Thermo Fisher Scientific Inc. (Waltham, MA, USA) according to the manufacturer's instructions. Subsequently, the supernatants were completely discarded and the cells were incubated with 1X PBS containing 10 *μ*M DCFDA at 37°C for 30 min. The intensity of 2,7-dichlorofluorescein (DCF) fluorescence was measured using the Synergy HT microplate reader (BioTek, Winooski, VT, USA) at excitation/emission wavelengths of 488/520 nm. The data were presented as the percentage of ROS relative to the untreated control.

### 2.6. Measurement of Mitochondrial Membrane Potential (ΔΨm)

The ΔΨm was measured using tetraethylbenzimidazolyl-carbocyanine iodide (JC-1) as the indicator of mitochondrial damage (Thermo Fisher Scientific Inc., Waltham, MA, USA). Briefly, SH-SY5Y cells were seeded into 96-well plates at a density of 2 × 10^4^ cells/well and treated as previously described above. After the treatment, cells were incubated with 5 *μ*M JC-1 at 37°C for 30 min. Cells were then gently washed twice with 1X PBS and the fluorescence intensity was measured using the Synergy HT microplate reader (BioTek, Winooski, VT, USA). The wavelengths of excitation/emission for green-fluorescent monomers were set at 490/530 nm and at 525/590 nm for red fluorescent J-aggregates. The data were presented as the ratio of monomer/J-aggregates.

### 2.7. Senescence-Associated *β*-Galactosidase Staining (SA-*β*-Gal)

The cellular senescence was performed using the senescence *β*-galactosidase staining kit (Cell Signaling Technology, Inc., Danvers, MA, USA) according to the manufacturer's instructions. In brief, SH-SY5Y cells were seeded into 24-well plates at a density of 3 × 10^4^ cells/well and treated as previously described above. Cells were washed twice with 1X PBS and fixed in 4% (v/v) formaldehyde for 10 mins at room temperature. This was followed by washing with distilled water for three times and then staining with the *β*-galactosidase detection solution (pH 6.0) at 37°C in a non-CO_2_ incubator for 16 h. Cells were observed under an Axiocam 305 color, a phase-contrast microscope (ZEISS, Germany) and the bright blue-staining cells were considered as the *β*-galactosidase positive cells. Cells were eventually counted for three random vision fields and the calculation of the senescence rate was performed.

### 2.8. Measurement of Senescence-Associated Secretory Phenotype (SASP)

The SASP was quantified from culture supernatants using an enzyme-linked immunosorbent assay (ELISA) kit (R&D Systems, Minneapolis, MN, USA) according to the manufacturer's instructions. After the treatment, the particulates were removed by centrifugation at 10,000 × g at 4°C for 10 min and the supernatants were collected and frozen at −80°C until further analysis. The levels of cytokines (IL-6 and IL-8) were assessed using ELISA kits specific to humans (R&D system). The absorbance was measured at 450 nm following the manufacturer's reference using the Synergy HT microplate reader (BioTek, Winooski, VT, USA) and the results were expressed in ng/ml.

### 2.9. Western Blot Analysis

Western blot experiments were performed following protocols as described previously [32]. Briefly, SH-SY5Y cells were harvested and subsequently lysed in the radioimmunoprecipitation (RIPA) assay buffer, supplemented with the 1% protease inhibitor cocktail. After centrifugation at 2,000 × g for 15 minutes at 4°C, the protein concentration was determined using a bicinchoninic acid (BCA) protein assay kit from Thermo Fisher Scientific Inc. (Waltham, MA, USA). Equal amounts (60 *μ*g per sample) of proteins per were loaded on 10–15% SDS-polyacrylamide gel electrophoresis (SDS-PAGE) and run at 100 V for 10 min, followed by 120 V for the remainder of the separation. The gel was transferred onto a 0.22 *μ*m nitrocellulose membrane (Bio-Rad, Hercules, CA, USA) and run at 100 V for 90 min. After blocking the nonspecific binding site with 5% nonfat dry milk in Tris-buffered saline (TBS) containing 0.1% Tween-20 (TBS-T) for 1 h at room temperature, the membranes were probed with antibodies against DMT1 (dilution, 1 : 500), FPN1 (dilution, 1 : 1000), FT-L (dilution, 1 : 1000), p53 (dilution, 1 : 2000), p21 (dilution, 1 : 1000), mTORC1 (dilution, 1 : 2000), p-mTORC1 (dilution, 1 : 2000), and *β*-actin (dilution, 1 : 5000) overnight at 4°C, along with secondary antibody conjugated with horseradish peroxidase (HRP) for 1 h at room temperature. The protein bands were enhanced using the SuperSignal West Pico chemiluminescence detection kit (Thermo Fisher Scientific Inc., Waltham, MA, USA) and visualized using the ChemiDoc XRS + System. The blots were analyzed using Image Lab Software (Bio-Rad, Hercules, CA, USA) and normalized to the expression of *β*-actin.

### 2.10. Statistical Analysis

All values are presented as mean ± standard error of the mean (SEM) from at least three independent experiments performed in triplicate. Comparison between groups was analyzed by using one-way ANOVA with Bonferroni's tests. Differences were considered statistically significant at *P* < 0.05. All statistical analysis was performed using GraphPad Prism 8.0.2 (GraphPad Software, San Diego, CA, USA).

## 3. Results

### 3.1. Ebselen Improves SH-SY5Y Cell Viability against FAC-Induced Cytotoxicity

**Figure 2:**
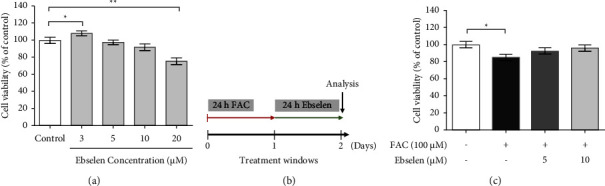
Ebselen improved viability in SH-SY5Y after the exposure to FAC. The cell viability was determined using the MTT assay. (a) Cell viability after the exposure to the serum-free medium containing various concentrations of ebselen for 24 h. (b) Ebselen treatment paradigm following FAC-induced intracellular iron accumulation. (c) Cell viability after the exposure to FAC (100 *μ*M) prior ebselen (5 and 10 *μ*M). The data are the mean ± SEM of the three independent experiments performed in triplicate. ^*∗*^*P* < 0.05 and ^*∗∗*^*P* < 0.01*vs*. the untreated group.

To determine whether the effective concentration of ebselen against the FAC-induced cytotoxicity and the viability of SH-SY5Y cells exposed solely to various concentrations of ebselen **(**3, 5, 10, and 20 *μ*M**)** were measured by the MTT assay. The viability of the cells treated with ebselen alone compared to the untreated control cells significantly decreased at the concentration of 20 *μ*M and promoted cell proliferation at the lowest concentration (3 *μ*M of ebselen) (Figure 2(a)). Therefore, the viability effect of 5 *μ*M and 10 *μ*M ebselen treatments were further investigated after the exposure to the 100 *μ*M FAC for 24 h (Figure 2(b)). The results showed that ebselen significantly improved the reduction of cell viability compared with the FAC-treated cells (Figure 2(c)). This highlighted the importance of ebselen in reducing SH-SY5Y cells' toxicity against FAC-induced cytotoxicity. Subsequently, the effective concentrations of FAC (100 *μ*M) and ebselen (5 and 10 *μ*M) were used in all experiments.

### 3.2. Downregulation of DMT1 Expression by Ebselen Enhances the Proper Iron Homeostasis in FAC-Treated SH-SY5Y Cells

The expression of iron uptake-mediated DMT1 in response to the varied exposure times of 100 *μ*M FAC (0, 6, 12, and 24 h) was determined in SH-SY5Y cells. The results showed that the cells treated with FAC for 24 h markedly upregulated DMT1 protein expression compared to the untreated control cells **(**Figures 1(a) and 1(b)) (see Supplementary data 1-2 for the original image of Figure 1(a)). Therefore, this exposure time (24 h) was selected for the examination of the effect of ebselen in the SH-SY5Y cells after 100 *μ*M FAC treatment. The results revealed that ebselen significantly decreased FAC-induced iron influx into the cell through DMT1 compared with that of the FAC-treated group (Figures 1(c) and 1(d)). The impact of ebselen on the key components in iron homeostasis in FAC-treated SH-SY5Y cells was also evaluated. FPN1, the only mammalian iron exporter identified to date [33] and FT-L, the major protein responsible for intracellular iron storage, were measured using Western blot analyses (see Supplementary data 3–6 for the original image of Figure 1(c)). FAC alone significantly reduced FPN1, but increased FT-L protein expressions compared with that of the untreated control cells. This was rescued following the ebselen treatment (Figures 1(b), 1(e), and 1(f)) which attenuated changes in the iron regulation. Moreover, ebselen also inhibits iron influx that may accelerate the extracellular iron transport.

### 3.3. Ebselen Reduces the Intracellular LIP Level in FAC-Induced Iron Overload in SH-SY5Y Cells

**Figure 3:**
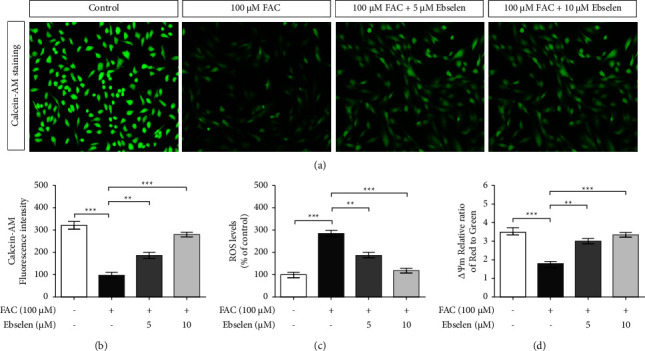
Ebselen reduced the intracellular iron level and oxidative stress following FAC-induced iron overload in SH-SY5Y cells. Cells were exposed to FAC (100 *μ*M) for 24 h prior to ebselen (5 and 10 *μ*M) for 24 h. The intracellular iron level based on the redox-active cytosolic LIP was examined using calcein-AM. (a) Representative images of cytosolic LIP were observed by using fluorescence microscope and taken at ×200 magnification. (b) The quantitative analysis of the intracellular iron level and the decreased calcein-AM fluorescence intensity indicate an increased intracellular iron level. (c) The quantitative analysis of the intracellular ROS level using DCFH-DA probe. (d) The quantitative analysis of ΔΨm using JC-1 dye. The data are the mean ± SEM of four independent experiments performed in triplicate. ^*∗∗∗*^*P* < 0.001 vs. the untreated group. ^*∗∗*^*P* < 0.01 and ^*∗∗∗*^*P* < 0.001*vs*. the FAC alone group.

The potential effects of ebselen on the cytosolic LIP level in FAC-treated SH-SY5Y cells were evaluated using the calcein-AM assay; and the reduction in calcein-AM fluorescence intensity signifies an increase in the cytosolic-chelatable Fe^2+^ [34]. The patches of green fluorescence were notably increased in the cytosol and diffused into lysosomes, where they were cleaved by the lysosomal esterase resulting in the calcein-AM penetration (Figure 3(a)). Therefore, the results indicated that the cytosolic LIP level significantly decreased following the ebselen treatment. However, this was rescued by the ebselen treatment (Figure 3(b)). These findings suggest that ebselen could directly inhibit iron influx and control the levels of cytosolic LIP in cultured SH-SY5Y cells.

### 3.4. Ebselen Suppresses ROS Accumulation and Rescues the Loss of ΔΨm by FAC-Induced Iron Overload in SH-SY5Y Cells

It is well documented that excessive iron is involved in an imbalance of redox homeostasis, resulting in the increase of intracellular ROS levels and oxidative stress [35]. This research investigated the antioxidative property of ebselen against FAC-induced intracellular ROS formation in SH-SY5Y cells using a DCFH-DA probe. The treatment with FAC alone significantly increased the intracellular ROS level compared with the untreated control cells. Interestingly, this was significantly decreased by ebselen treatment and the level was comparable to that in the control group (Figure 3(c)). These findings suggest that ebselen possesses antioxidant properties, which could potentially dampen the depolarization of mitochondrial membrane potential (ΔΨM) of aging neuronal cells caused by oxidative stress. The potential effects of ebselen on the rescuing of the oxidative stress-induced ΔΨm impairment were confirmed using the JC-1 probe. The results revealed a significant decrease in the ratio of green/red fluorescence intensity in the FAC-treated cells, which implied the impairment of ΔΨM. This was rescued by the ebselen treatment, resulting in the increase of the green/red fluorescence intensity ratio (Figure 3(d)).

### 3.5. Ebselen Restores SH-SY5Y Senescence via Inhibiting the mTORC1 Signaling Pathway

**Figure 4:**
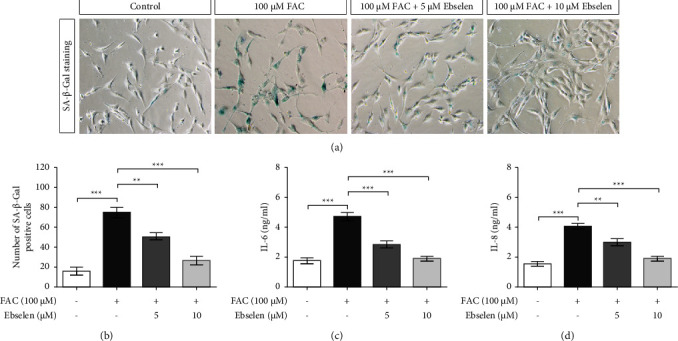
Ebselen rescued iron overload-induced neuronal senescence. Cells were exposed to FAC (100 *μ*M) for 24 h prior to ebselen (5 and 10 *μ*M) for 24 h and then the senescence markers were measured. (a) Representative images of SH-SY5Y stained with SA-*β*-gal, the images were obtained by phase-contrast microscopy, and taken at ×200 magnification. (b) The percentage of SA-*β*-gal-positive cells per field and three vision fields from each replica were scored. The amount of SASP secretion, (c) IL-6, and (d) IL-8, was analyzed using ELISA. The data are the mean ± SEM of the three independent experiments performed in triplicate. ^*∗∗∗*^*P* < 0.001 vs. the untreated group. ^*∗∗*^*P* < 0.01 and ^*∗∗∗*^*P* < 0.001*vs*. the FAC alone group.

**Figure 5:**
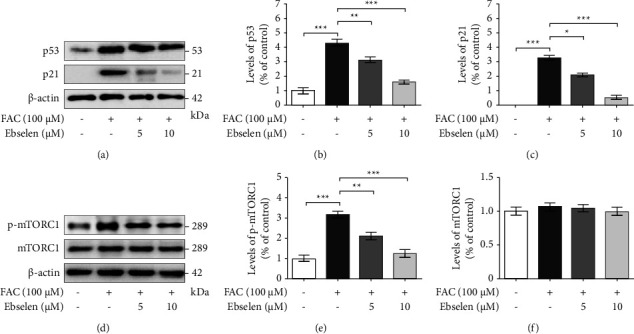
Ebselen counteracts neuronal senescence through the mTORC1 pathway. Cells were exposed to FAC (100 *μ*M) for 24 h prior to ebselen (5 and 10 *μ*M) for 24 h and then the expression of markers for the cell cycle was measured. (a) Cell cycle checkpoint protein p53/cyclin-dependent kinase (CDK) inhibitors p21 were evaluated using the Western blot analysis. The quantitative analysis of the expression of (b) p53 and (c) p21 was normalized using *β*-actin, respectively. (d) Western blot analysis of phosphorylated p-mTORC1 and total mTORC1. The quantitative analysis of the expression of (e) p-mTORC1 and (f) total mTORC1 was normalized using *β*-actin, respectively. The data are the mean ± SEM of the three independent experiments performed in triplicate. ^*∗∗∗*^*P* < 0.001 vs. the untreated group. ^*∗*^*P* < 0.05, ^*∗∗*^*P* < 0.01, and ^*∗∗∗*^*P* < 0.001*vs*. the FAC alone group (see supplementary data 7–9 for the original image of Figure 5(a) and supplementary data 10–12 for the original image of Figure 5(d), respectively).

The potential mechanisms of ebselen in restoring the iron overload-associated senescence and oxidative stress in SH-SY5Y cells were evaluated using a collection of cellular senescence markers including SA-*β*-gal, SASP, and cell cycle inhibitors activated by DNA damage response (DDR). This is due to the fact that senescent feature alone is insufficient to determine a senescent state. The iron overload in the SH-SY5Y cells is firmly associated with an increased number of SA-*β*-gal positive cells compared with the untreated control cells, which were almost unaffected. In contrast, the SA-*β*-gal positive cells were partially reverted following the ebselen treatment, particularly at a concentration of 10 *μ*M (Figures 4(a) and 4(b)). Since SASP has been shown to induce paracrine senescence and chronic inflammation in the aging brain, the expression of a common SASP component, including IL-6 and IL-8, using ELISA was performed. The results revealed that there was a marked increase in IL-6 and IL-8 proteins in the senescent SH-SY5Y culture media, which was significantly reduced after the ebselen treatment (Figures 4(c) and 4(d)). Furthermore, the iron overload and the overproduction of ROS increased the activation of p53 and the expression of downstream proteins p21, which were effectively reversed by ebselen (Figures 5(a)–5(c)) (see Supplementary data 7–9 for the original image of Figure 5(a)). The investigation of mTORC1 activity was also carried out and the results showed that the oxidative stress induced the phosphorylation of mTORC1. Conversely, ebselen significantly downregulated the phosphorylation of mTORC1 (Figures 5(d)–5(f)), demonstrating that iron overload creates neuronal senescence through the activation of the mTORC1 signaling pathway (see Supplementary data 10–12 for the original image of Figure 5(d)).

## 4. Discussion

Iron accumulation is the function of aging as it plays a pivotal role in activating a cascade of pathophysiological processes in cellular senescence via oxidative stress. Ebselen, a restoring antiaging agent, was investigated in the present study to determine its restoring potential in the iron overload-induced senescence-like neuronal cells, SH-SY5Y.

Iron overload in the brain amplifies neuronal toxicity through its redox-active effects, leading to cellular senescence, which preferentially occurs in physiological brain aging and corresponds to the severity of neurodegenerative diseases, especially AD and PD lesions [36, 37]. Given the last decade of clinical trials, drug therapeutic for AD has largely been directed at the A*β* peptide, owing to the hypothesis of disease that it is a critical initiator of the progression of lesions [38]. However, medication to modify the pathogenesis of AD has not yet been identified. Ebselen, on the other hand, is a widely recognized synthetic peroxynitrite scavenger compound that mimics the activity of GPx, which catalyzes essential reactions that provide protection against oxidative damage [24]. Ebselen has been widely used as a therapeutic agent in the clinical trials of various diseases [39, 40]. A recent study demonstrated that ebselen acts as a potent anti-inflammatory agent by suppressing the TRAF2-ASK1-SEK1 signaling pathway, which is essential for endoplasmic reticulum stress-induced cell death [41], and is consistent with the fact that ebselen has neuropharmacological properties, which possess antioxidant and anti-inflammatory activity and free radicals scavenging [42]. In addition, dyshomeostasis of brain iron causes aggregation of A*β* peptides and tau protein, consequently promoting neuronal cytotoxicity, which is a pathological hallmark of AD [43, 44]. Several studies have revealed the potential effects of ebselen in preventing the progression of AD-related pathology and enhancing the spatial learning and memory in two-month-old 3 × Tg-AD mice, suggesting that ebselen could be a potential therapeutic agent for the prevention or treatment of AD [45]. Furthermore, ebselen also has multifunctional properties, including reversing oxidative stress, neuronal apoptosis, and memory impairment in a sporadic model of AD induced by intracerebroventricular administration of streptozocin [27]. These findings imply that ebselen could be a therapeutic target for AD. Iron has also been shown to enhance ferroptosis, a type of regulated necrosis characterized by iron-induced lipid peroxidation that causes catastrophic membrane rupture, which is controlled by GPx4, an event that occurs in AD progression [46]. Due to the GPX mimetic properties, the selenium compound ebselen could reduce neuronal ferroptosis in traumatic brain injury with cerebral ischemia and intracerebral hemorrhage, respectively [47, 48]. Nonetheless, there are few studies elucidating the benefits of ebselen as a treatment strategy for curing brain iron overload-associated senescence-like neuronal cells that accelerate the development of AD.

The mechanism underlying iron progressively accumulating in senescent cells has not been fully understood. We first examined the potential of ebselen to alleviate the dysregulation of iron homeostasis in SH-SY5Y cells following the exposure to FAC. Ebselen has been identified as a DMT1 inhibitor, which plays a vital role in direct DMT1 transport blockage and iron-catalyzed damage limitation [28]. This could reverse the dopaminergic neuronal loss and motoric deficit in the brain regions implicated in PD [49]. Neuronal iron transport is principally dependent on the NTBI transport pathway via DMT1 [4]. Our results demonstrated that ebselen significantly decreased DMT1 expression upon the increase of intracellular iron level, indicating that the high-affinity property of iron chelator inhibits iron uptake and pumps iron into the cytosol [31, 50]. In addition, FAC-induced dysregulation of iron homeostasis was considerably mitigated by ebselen. Since ebselen increased FPN1 expression, while decreasing the expression of FT-L and the cytosolic LIP levels; it might exert critical routes to DMT1 inhibitor-mediated FPN1 degradation that plays an essential role in the export of iron. Possibly, ebselen may chelate labile iron to form a relatively stable compound to maintain iron homeostasis [29, 30].

The collective evidence shows that Fe^2+^ is an effective modulator to catalyze the conversion of H_2_O_2_ to hydroxyl radicals via the Fenton reaction [5]. This is in line with many studies which have reported that increased LIP leads to a marked increase in ROS formation, the key propagation to DNA damage and mitochondrial function decline observed in mammalian aging [51, 52]. Our results showed that ebselen markedly decreased intracellular ROS levels and increased ΔΨm following the iron overload in SH-SY5Y cells. These outcomes provide a strong evidence supporting the direct property of ebselen as a powerful antioxidant and radical scavenger in the ischemia/reperfusion model [25]. As a consequence of the extensive oxidative injury, cells could either react toward a defensive mechanism or the pathological process, leading to mitochondrial dysfunction. This is apparently distinct in the accumulation of senescent cells and secretory phenotype [53, 54].

Despite being postmitotic cells, neurons could be capable of undergoing senescence in response to oxidative stress [55]. Further experiments must be carried out to evaluate whether iron accumulation might be a critical factor in brain aging and to assess the property of ebselen on antiaging in reliable neuronal senescence. The molecular mechanism responsible for cellular senescence demonstrated the downregulation of the nuclear receptor coactivator 4 (NCOA4), a selective cargo receptor for autophagic turnover of ferritin, which induces ferritin accumulation and increases DNA instability and lysosomal dysfunction [56, 57]. Consequently, senescent cells likely accumulate iron partially due to failure to degrade ferritin [58], thereby sequestering iron in an inactive state and leading to cell-cycle arrest [23]. Besides, SA-*β*-Gal is the most widely used marker of senescence derived from lysosomal enzymes and can be detected at early stages. It is well established that excessive ROS strongly triggers cellular senescence via regulating multiple pathways. Proinflammatory cytokines, termed senescence-associated secretory phenotype (SASP), can increase c-Jun N-terminal kinase (JNK) phosphorylation [59] and activate the ROS/NF-*κ*B pathway [30], thus accelerating cellular senescence. The SASP paracrine signaling is a crucial pathological feature of cellular senescence that causes local chronic inflammation [18, 60]. Not only glial cells are responsible for releasing proinflammatory cytokines, but also neurons, resulting in a self-propelling cycle of neuroinflammation and eventually neurodegeneration [61]. Our results demonstrated that ebselen could restore neuronal senescence by significantly reducing *β*-gal activity and decreasing the expression of IL-6 and IL-8 levels, which is related to its potent anti-inflammatory properties [62].

In addition, the appearance of senescence phenotype in our study demonstrated that neurons respond to the damage by activating the p53 pathway and its downstream, p21, known as CDKN1A (cyclin-dependent kinase inhibitor 1) [17, 18]. Phosphorylation of p53 has been shown to be critical for telomeric stress-induced cellular senescence and is closely linked to DNA double-stranded breaks (DSBs) and DNA damage response (DDR) activation [63]. Ebselen notably restored neuronal senescence by the downregulation of p53/p21 expression. The results provide evidence that ebselen could decrease the acceleration of senescence through the reduction of ROS levels. By simultaneously investigating the signaling pathway involved in cellular senescence in parallel experiments, we found that ebselen could induce the downregulation of mTORC1 expression, a key mediator of cellular senescence. It has been reported that oxidative stress-dependent mitochondrial depletion exacerbates the proaging phenotype thereby promoting the mTOR phosphorylation cascade [64]. Therefore, the inhibition of the mTORC1 pathway by ebselen might restore iron overload-induced senescence-like neuronal cells, SH-SY5Y, by suppressing the SASP regulatory proteins, thereby improving the mitochondrial integrity and reversing cell cycle arrest.

## 5. Conclusions

**Figure 6:**
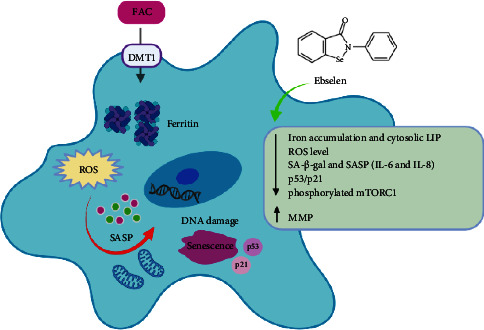
A schematic model representation of the proposed mechanism of ebselen on FAC-induced iron overload associated with senescent-like neuronal cells, SH-SY5Y. FAC facilitates an increased intracellular iron levels via DMT1, the main pathway responsible for cellular iron uptake in neurons. Subsequently, increasing intracellular ROS formation via the Fenton reaction that mediates proinflammatory cytokine release leads to the damage of mitochondrial membrane and DNA. Excessive intracellular iron levels are undoubtedly one of the main causes of cellular senescence, leading to organismal aging. Ebselen is a DMT1 inhibitor that could decrease intracellular iron levels by increasing the expression of FPN1 iron exporter and decreasing FT-L iron storage. Furthermore, ebselen could rescue neuronal senescence by reducing ROS levels and by improving ΔΨm and cellular senescence markers, including SA-*β*-gal, SASP profile, and p53/p21 via inhibiting the mTORC1 signaling pathway.

We conclude that excessive iron plays a pivotal role in accelerating the processes of brain aging, especially neurodegenerative diseases. Our study provides evidence that ebselen, a DMT1 inhibitor, might be one of the therapeutic options for mitigating iron overload-associated senescence-like neuronal cells, SH-SY5Y, through direct and indirect antioxidative and anti-inflammatory activities (Figure 6). While we find that these studies offer significant insights that contribute to the scientific community's ongoing interest, it remains crucial to recognize that our current work is merely the initial step. The efficacy of ebselen appears to prompt the need for subsequent *in vivo* studies, which we anticipate will be undertaken in the near future. These studies will likely pave the way for eventual clinical trials, constituting a critical phase in uncovering the full potential of ebselen as a prospective neurodegenerative medicine. In our perspective, our present research and the forthcoming studies will collectively advance our understanding of ebselen's therapeutic value and contribute to addressing the challenges posed by neurodegenerative diseases. The combination of *in vivo* investigations and clinical trials will provide deeper insights into ebselen's efficacy and its potential impact as a future therapeutic medicine.

## Data Availability

The data used to support the findings of this study are included within the article.

## Supplementary Materials

Supplementary Materials Supplementary data 1: original image of divalent metal transporter 1 (DMT1) blot for Figure 1(a). Supplementary data 2: original image of *β*-actin blot for Figure 1(a). Supplementary data 3: original image of divalent metal transporter 1 (DMT1) blot for Figure 1(c). Supplementary data 4: original image of ferroportin 1 (FPN1) blot for Figure 1(c). Supplementary data 5: original image of ferritin light chain (FT-L) blot for Figure 1(c). Supplementary data 6: original image of *β*-actin blot for Figure 1(c). Supplementary data 7: original image of p53 blot for Figure 5(a). Supplementary data 8: original image of p21 blot for Figure 5(a). Supplementary data 9: original image of *β*-actin blot for Figure 5(a). Supplementary data 10: original image of p-mTORC1 blot for Figure 5(d). Supplementary data 11: original image of mTORC1 blot for Figure 5(d). Supplementary data 12: original image of *β*-actin blot for Figure 5(d).
