# Supplementary figures and images for: Ebselen, Iron Uptake Inhibitor, Alleviates Iron Overload-Induced Senescence-Like Neuronal Cells SH-SY5Y via Suppressing the mTORC1 Signaling Pathway
Source: Adv Pharmacol Pharm Sci. 2023 Sep 12;2023:6641347. doi: 10.1155/2023/6641347 (PMC10509000; doi:10.1155/2023/6641347)

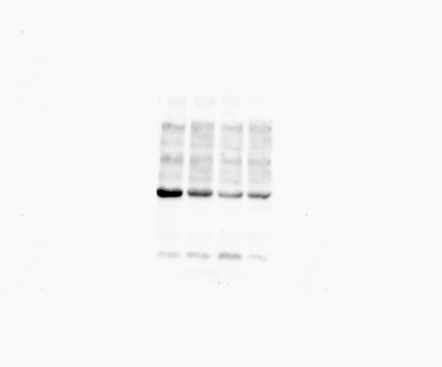

Supplement: Supplementary Materials — Supplementary data 1: original image of divalent metal transporter 1 (DMT1) blot for Figure 1(a). Supplementary data 2: original image of β-actin blot for Figure 1(a). Supplementary data 3: original image of divalent metal transporter 1 (DMT1) blot for Figure 1(c). Supplementary data 4: original image of ferroportin 1 (FPN1) blot for Figure 1(c). Supplementary data 5: original image of ferritin light chain (FT-L) blot for Figure 1(c). Supplementary data 6: original image of β-actin blot for Figure 1(c). Supplementary data 7: original image of p53 blot for Figure 5(a). Supplementary data 8: original image of p21 blot for Figure 5(a). Supplementary data 9: original image of β-actin blot for Figure 5(a). Supplementary data 10: original image of p-mTORC1 blot for Figure 5(d). Supplementary data 11: original image of mTORC1 blot for Figure 5(d). Supplementary data 12: original image of β-actin blot for Figure 5(d). [file 6641347.f1.zip › Supplementary data 1 (1).docx]

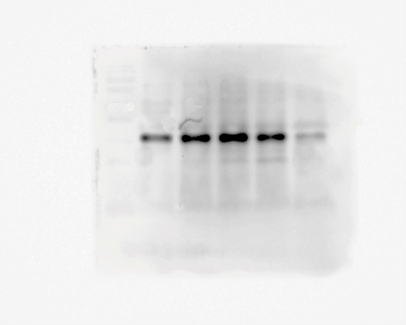

Supplement: Supplementary Materials — Supplementary data 1: original image of divalent metal transporter 1 (DMT1) blot for Figure 1(a). Supplementary data 2: original image of β-actin blot for Figure 1(a). Supplementary data 3: original image of divalent metal transporter 1 (DMT1) blot for Figure 1(c). Supplementary data 4: original image of ferroportin 1 (FPN1) blot for Figure 1(c). Supplementary data 5: original image of ferritin light chain (FT-L) blot for Figure 1(c). Supplementary data 6: original image of β-actin blot for Figure 1(c). Supplementary data 7: original image of p53 blot for Figure 5(a). Supplementary data 8: original image of p21 blot for Figure 5(a). Supplementary data 9: original image of β-actin blot for Figure 5(a). Supplementary data 10: original image of p-mTORC1 blot for Figure 5(d). Supplementary data 11: original image of mTORC1 blot for Figure 5(d). Supplementary data 12: original image of β-actin blot for Figure 5(d). [file 6641347.f1.zip › Supplementary data 10.docx]

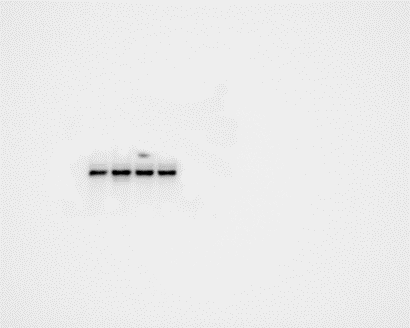

Supplement: Supplementary Materials — Supplementary data 1: original image of divalent metal transporter 1 (DMT1) blot for Figure 1(a). Supplementary data 2: original image of β-actin blot for Figure 1(a). Supplementary data 3: original image of divalent metal transporter 1 (DMT1) blot for Figure 1(c). Supplementary data 4: original image of ferroportin 1 (FPN1) blot for Figure 1(c). Supplementary data 5: original image of ferritin light chain (FT-L) blot for Figure 1(c). Supplementary data 6: original image of β-actin blot for Figure 1(c). Supplementary data 7: original image of p53 blot for Figure 5(a). Supplementary data 8: original image of p21 blot for Figure 5(a). Supplementary data 9: original image of β-actin blot for Figure 5(a). Supplementary data 10: original image of p-mTORC1 blot for Figure 5(d). Supplementary data 11: original image of mTORC1 blot for Figure 5(d). Supplementary data 12: original image of β-actin blot for Figure 5(d). [file 6641347.f1.zip › Supplementary data 11.docx]

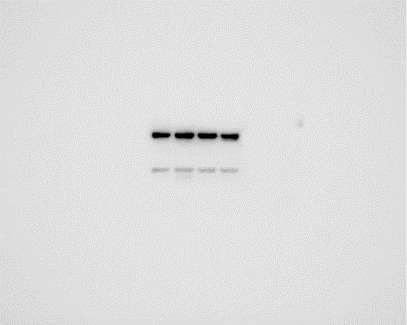

Supplement: Supplementary Materials — Supplementary data 1: original image of divalent metal transporter 1 (DMT1) blot for Figure 1(a). Supplementary data 2: original image of β-actin blot for Figure 1(a). Supplementary data 3: original image of divalent metal transporter 1 (DMT1) blot for Figure 1(c). Supplementary data 4: original image of ferroportin 1 (FPN1) blot for Figure 1(c). Supplementary data 5: original image of ferritin light chain (FT-L) blot for Figure 1(c). Supplementary data 6: original image of β-actin blot for Figure 1(c). Supplementary data 7: original image of p53 blot for Figure 5(a). Supplementary data 8: original image of p21 blot for Figure 5(a). Supplementary data 9: original image of β-actin blot for Figure 5(a). Supplementary data 10: original image of p-mTORC1 blot for Figure 5(d). Supplementary data 11: original image of mTORC1 blot for Figure 5(d). Supplementary data 12: original image of β-actin blot for Figure 5(d). [file 6641347.f1.zip › Supplementary data 12.docx]

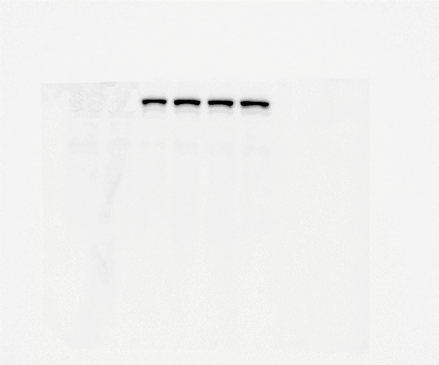

Supplement: Supplementary Materials — Supplementary data 1: original image of divalent metal transporter 1 (DMT1) blot for Figure 1(a). Supplementary data 2: original image of β-actin blot for Figure 1(a). Supplementary data 3: original image of divalent metal transporter 1 (DMT1) blot for Figure 1(c). Supplementary data 4: original image of ferroportin 1 (FPN1) blot for Figure 1(c). Supplementary data 5: original image of ferritin light chain (FT-L) blot for Figure 1(c). Supplementary data 6: original image of β-actin blot for Figure 1(c). Supplementary data 7: original image of p53 blot for Figure 5(a). Supplementary data 8: original image of p21 blot for Figure 5(a). Supplementary data 9: original image of β-actin blot for Figure 5(a). Supplementary data 10: original image of p-mTORC1 blot for Figure 5(d). Supplementary data 11: original image of mTORC1 blot for Figure 5(d). Supplementary data 12: original image of β-actin blot for Figure 5(d). [file 6641347.f1.zip › Supplementary data 2 (1).docx]

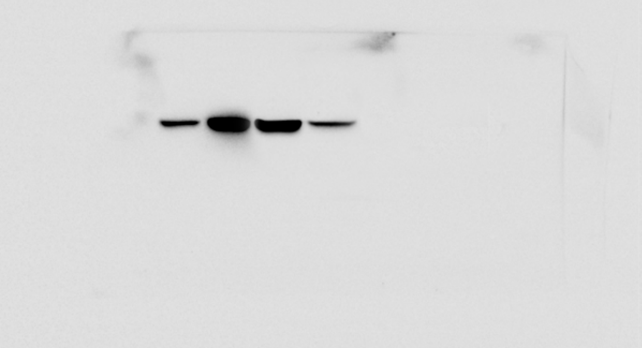

Supplement: Supplementary Materials — Supplementary data 1: original image of divalent metal transporter 1 (DMT1) blot for Figure 1(a). Supplementary data 2: original image of β-actin blot for Figure 1(a). Supplementary data 3: original image of divalent metal transporter 1 (DMT1) blot for Figure 1(c). Supplementary data 4: original image of ferroportin 1 (FPN1) blot for Figure 1(c). Supplementary data 5: original image of ferritin light chain (FT-L) blot for Figure 1(c). Supplementary data 6: original image of β-actin blot for Figure 1(c). Supplementary data 7: original image of p53 blot for Figure 5(a). Supplementary data 8: original image of p21 blot for Figure 5(a). Supplementary data 9: original image of β-actin blot for Figure 5(a). Supplementary data 10: original image of p-mTORC1 blot for Figure 5(d). Supplementary data 11: original image of mTORC1 blot for Figure 5(d). Supplementary data 12: original image of β-actin blot for Figure 5(d). [file 6641347.f1.zip › Supplementary data 3 (1).docx]

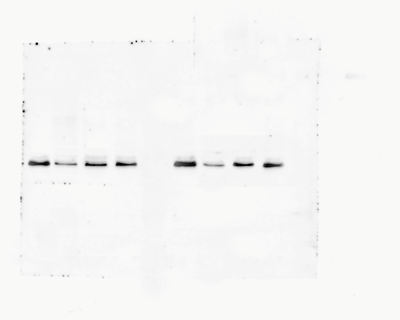

Supplement: Supplementary Materials — Supplementary data 1: original image of divalent metal transporter 1 (DMT1) blot for Figure 1(a). Supplementary data 2: original image of β-actin blot for Figure 1(a). Supplementary data 3: original image of divalent metal transporter 1 (DMT1) blot for Figure 1(c). Supplementary data 4: original image of ferroportin 1 (FPN1) blot for Figure 1(c). Supplementary data 5: original image of ferritin light chain (FT-L) blot for Figure 1(c). Supplementary data 6: original image of β-actin blot for Figure 1(c). Supplementary data 7: original image of p53 blot for Figure 5(a). Supplementary data 8: original image of p21 blot for Figure 5(a). Supplementary data 9: original image of β-actin blot for Figure 5(a). Supplementary data 10: original image of p-mTORC1 blot for Figure 5(d). Supplementary data 11: original image of mTORC1 blot for Figure 5(d). Supplementary data 12: original image of β-actin blot for Figure 5(d). [file 6641347.f1.zip › Supplementary data 4 (1).docx]

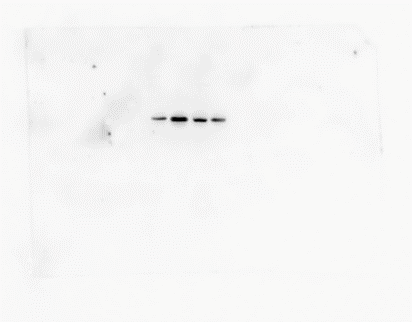

Supplement: Supplementary Materials — Supplementary data 1: original image of divalent metal transporter 1 (DMT1) blot for Figure 1(a). Supplementary data 2: original image of β-actin blot for Figure 1(a). Supplementary data 3: original image of divalent metal transporter 1 (DMT1) blot for Figure 1(c). Supplementary data 4: original image of ferroportin 1 (FPN1) blot for Figure 1(c). Supplementary data 5: original image of ferritin light chain (FT-L) blot for Figure 1(c). Supplementary data 6: original image of β-actin blot for Figure 1(c). Supplementary data 7: original image of p53 blot for Figure 5(a). Supplementary data 8: original image of p21 blot for Figure 5(a). Supplementary data 9: original image of β-actin blot for Figure 5(a). Supplementary data 10: original image of p-mTORC1 blot for Figure 5(d). Supplementary data 11: original image of mTORC1 blot for Figure 5(d). Supplementary data 12: original image of β-actin blot for Figure 5(d). [file 6641347.f1.zip › Supplementary data 5.docx]

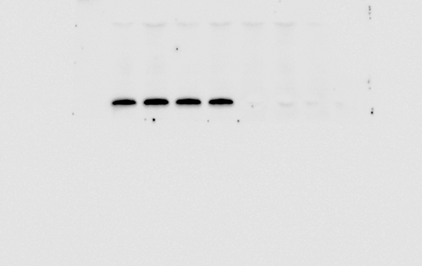

Supplement: Supplementary Materials — Supplementary data 1: original image of divalent metal transporter 1 (DMT1) blot for Figure 1(a). Supplementary data 2: original image of β-actin blot for Figure 1(a). Supplementary data 3: original image of divalent metal transporter 1 (DMT1) blot for Figure 1(c). Supplementary data 4: original image of ferroportin 1 (FPN1) blot for Figure 1(c). Supplementary data 5: original image of ferritin light chain (FT-L) blot for Figure 1(c). Supplementary data 6: original image of β-actin blot for Figure 1(c). Supplementary data 7: original image of p53 blot for Figure 5(a). Supplementary data 8: original image of p21 blot for Figure 5(a). Supplementary data 9: original image of β-actin blot for Figure 5(a). Supplementary data 10: original image of p-mTORC1 blot for Figure 5(d). Supplementary data 11: original image of mTORC1 blot for Figure 5(d). Supplementary data 12: original image of β-actin blot for Figure 5(d). [file 6641347.f1.zip › Supplementary data 6.docx]

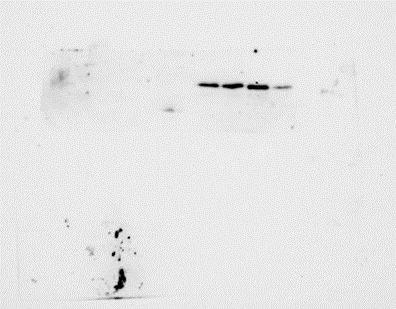

Supplement: Supplementary Materials — Supplementary data 1: original image of divalent metal transporter 1 (DMT1) blot for Figure 1(a). Supplementary data 2: original image of β-actin blot for Figure 1(a). Supplementary data 3: original image of divalent metal transporter 1 (DMT1) blot for Figure 1(c). Supplementary data 4: original image of ferroportin 1 (FPN1) blot for Figure 1(c). Supplementary data 5: original image of ferritin light chain (FT-L) blot for Figure 1(c). Supplementary data 6: original image of β-actin blot for Figure 1(c). Supplementary data 7: original image of p53 blot for Figure 5(a). Supplementary data 8: original image of p21 blot for Figure 5(a). Supplementary data 9: original image of β-actin blot for Figure 5(a). Supplementary data 10: original image of p-mTORC1 blot for Figure 5(d). Supplementary data 11: original image of mTORC1 blot for Figure 5(d). Supplementary data 12: original image of β-actin blot for Figure 5(d). [file 6641347.f1.zip › Supplementary data 7.docx]

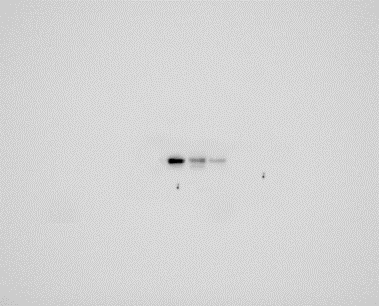

Supplement: Supplementary Materials — Supplementary data 1: original image of divalent metal transporter 1 (DMT1) blot for Figure 1(a). Supplementary data 2: original image of β-actin blot for Figure 1(a). Supplementary data 3: original image of divalent metal transporter 1 (DMT1) blot for Figure 1(c). Supplementary data 4: original image of ferroportin 1 (FPN1) blot for Figure 1(c). Supplementary data 5: original image of ferritin light chain (FT-L) blot for Figure 1(c). Supplementary data 6: original image of β-actin blot for Figure 1(c). Supplementary data 7: original image of p53 blot for Figure 5(a). Supplementary data 8: original image of p21 blot for Figure 5(a). Supplementary data 9: original image of β-actin blot for Figure 5(a). Supplementary data 10: original image of p-mTORC1 blot for Figure 5(d). Supplementary data 11: original image of mTORC1 blot for Figure 5(d). Supplementary data 12: original image of β-actin blot for Figure 5(d). [file 6641347.f1.zip › Supplementary data 8.docx]

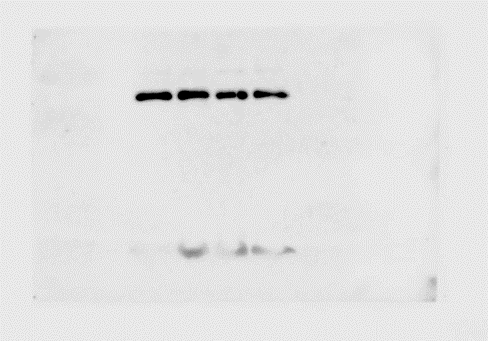

Supplement: Supplementary Materials — Supplementary data 1: original image of divalent metal transporter 1 (DMT1) blot for Figure 1(a). Supplementary data 2: original image of β-actin blot for Figure 1(a). Supplementary data 3: original image of divalent metal transporter 1 (DMT1) blot for Figure 1(c). Supplementary data 4: original image of ferroportin 1 (FPN1) blot for Figure 1(c). Supplementary data 5: original image of ferritin light chain (FT-L) blot for Figure 1(c). Supplementary data 6: original image of β-actin blot for Figure 1(c). Supplementary data 7: original image of p53 blot for Figure 5(a). Supplementary data 8: original image of p21 blot for Figure 5(a). Supplementary data 9: original image of β-actin blot for Figure 5(a). Supplementary data 10: original image of p-mTORC1 blot for Figure 5(d). Supplementary data 11: original image of mTORC1 blot for Figure 5(d). Supplementary data 12: original image of β-actin blot for Figure 5(d). [file 6641347.f1.zip › Supplementary data 9.docx]
